# Invited review: Translating kisspeptin and neurokinin B biology into new therapies for reproductive health

**DOI:** 10.1111/jne.13201

**Published:** 2022-10-19

**Authors:** Edouard G. Mills, Waljit S. Dhillo

**Affiliations:** ^1^ Section of Endocrinology and Investigative Medicine Imperial College London London UK; ^2^ Department of Endocrinology Imperial College Healthcare NHS Trust London UK

**Keywords:** fertility, kisspeptin, neurokinin B, reproduction

## Abstract

The reproductive neuropeptide kisspeptin has emerged as the master regulator of mammalian reproduction due to its key roles in the initiation of puberty and the control of fertility. Alongside the tachykinin neurokinin B and the endogenous opioid dynorphin, these peptides are central to the hormonal control of reproduction. Building on the expanding body of experimental animal models, interest has flourished with human studies revealing that kisspeptin administration stimulates physiological reproductive hormone secretion in both healthy men and women, as well as patients with common reproductive disorders. In addition, emerging therapeutic roles based on neurokinin B for the management of menopausal flushing, endometriosis and uterine fibroids are increasingly recognised. In this review, we focus on kisspeptin and neurokinin B and their potential application as novel clinical strategies for the management of reproductive disorders.

## Graphical abstract

Schematic diagram illustrating the potential clinical benefits of manipulating kisspeptin and neurokinin‐B signalling.

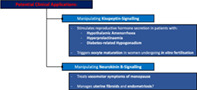

## 1 INTRODUCTION

Reproductive disorders are a broad range of highly prevalent conditions, frequently caused by aberrations in the hypothalamic‐pituitary‐gonadal (HPG) reproductive axis. Indeed, recent epidemiological data reveals that infertility is experienced by 12.5% of women and 10.1% of men in the United Kingdom.^1^ Despite the major health burden associated with reproductive disorders, many standard therapies are limited by poor efficacy, contraindicated in many and carry significant side‐effects. Novel safe and effective clinical strategies are therefore much needed.

The reproductive neuropeptide kisspeptin has emerged as critical for mammalian reproduction due to its key role as an upstream regulator of gonadotropin‐releasing hormone (GnRH) secretion, thereby coordinating the hypothalamic‐pituitary‐gonadal (HPG) axis.^2^ Alongside the tachykinin neurokinin B (NKB) and the endogenous opioid dynorphin (Dyn), these peptides are central to the hormonal control of reproduction. Given their importance, research has flourished in the field of kisspeptin, neurokinin B and dynorphin biology, paving the way to the development of therapeutics based on these peptides. In this review, we focus on the experimental animal data and human studies examining kisspeptin in health and disease. In addition, we summarise emerging therapeutic roles for neurokinin B in the management of menopausal flushing, endometriosis and uterine fibroids. A comprehensive and up‐to‐date understanding of this important area in reproductive biology is key to the development of novel clinical strategies for the management of reproductive disorders.

## 2 THE REPRODUCTIVE AXIS: A HISTORICAL PERSPECTIVE

Reproduction and fertility are controlled by the HPG axis, which acts as a tightly regulated neuroendocrine network to control gonadal development and function. Despite the fundamental role of kisspeptin‐signalling in reproductive function, early study focused on its involvement in cancer biology when in 1996 the kisspeptin gene (*KISS1* in humans and *Kiss1* in nonhumans) was first discovered as a metastasis suppressor gene in human melanoma cell lines.^3^ In 1999 the gene encoding a G protein‐coupled receptor was identified and initially termed G protein‐coupled receptor 54 (GPR54).^4^ In 2001 it was redesignated the “kisspeptin receptor” (KISS1R in humans and Kiss1r in nonhumans) following demonstration by independent groups that the *KISS1/Kiss1* gene product “metastin” (now known as kisspeptin‐54) acted as its natural ligand.^5^, ^6^, ^7^ Along with the shorter peptides (kisspeptin‐14, ‐13, and ‐10) derived from the same precursor protein, they became known as kisspeptin.

Shortly after discovery, evidence emerged of unambiguous physiological functions extending beyond cancer biology. In 2003 two separate groups published reports in rapid succession describing individuals from two consanguineous families with hypogonadotropic hypogonadism (HH) due to an inactivating mutation of *KISS1R*.^8^, ^9^ Concurrently, an equivalent reproductive phenotype was recapitulated in mice by targeted deletion of *Kiss1r*.^9^ Subsequent key supporting findings included demonstration that mice and humans with inactivating mutations in the *KISS1/Kiss1* gene failed to undergo normal sexual maturation.^10^, ^11^ Conversely, activating mutations of *KISS1R* in humans caused central precocious puberty.^12^ Following the discovery of kisspeptin and its receptor, a subpopulation of neurons was identified within the hypothalamic arcuate nucleus of several mammalian species in which kisspeptin is colocalised with NKB and Dyn.^13^, ^14^, ^15^, ^16^ Taken together, these historic discoveries in reproductive biology have been critical in identifying kisspeptin as essential for reproductive health.

## 3 KISSPEPTIN ISOFORMS

The kisspeptin isoforms are structurally related peptides encoded by the *KISS1/Kiss1* gene. Differing in amino acid length, they are the proteolytic products of a common 145‐amino acid precursor, resulting in four fragments: kisspeptin‐54, −14, −13 and − 10 (suffix conveying the amino acid length).^5^, ^6^ The major circulating isoform in humans is believed to be kisspeptin‐54 as suggested by data in pregnancy.^17^ However, the degree to which each isoform is present in the circulation remains unknown.^18^ Each isoform shares a common C‐terminal decapeptide sequence (corresponding to kisspeptin‐10), which confers biological activity at the kisspeptin receptor.^5^, ^6^ Furthermore, competition binding experiments reveal that the kisspeptin isoforms are equipotent on both the rat and human kisspeptin receptors, suggesting that each isoform has a similar affinity and efficacy for the kisspeptin receptor in vitro.^6^ Hence, this indicates that the C‐terminal decapeptide sequence is responsible for the high binding affinity and activation of the kisspeptin receptor.^6^

Regarding in vivo properties, administration of kisspeptin‐54 demonstrates more potent effects than kisspeptin‐10, which probably reflects the ability of kisspeptin‐54 (but probably not kisspeptin‐10) to cross the blood–brain barrier.^19^ Additionally, intraperitoneal administration of kisspeptin to male mice has been observed to rapidly access the bloodstream with kisspeptin‐10 reaching maximum levels after 2 min and kisspeptin‐54 after 5 min.^19^ In this experimental model, despite administration of equimolar amounts, peak levels of kisspeptin‐54 in the bloodstream were almost 50 times higher than after administration of kisspeptin‐10.^19^ In addition, peak plasma kisspeptin levels lasted until 30 min post‐administration of kisspeptin‐54 followed by progressive diminishment until 120 min, whereas plasma kisspeptin levels were undetectable by 10‐min following kisspeptin‐10 administration,^19^ demonstrating a longer profile for kisspeptin‐54. Indeed, in healthy men the circulating half‐life of kisspeptin‐54 is 27.6 (±1.1) min.^20^ In contrast, the circulating half‐life of kisspeptin‐10 is significantly shorter at 3.8 (±0.3) and 4.1 (±0.4) min in healthy men and women, respectively, reflecting increased proteolytic degradation in circulation.^21^ These pharmacological differences have important implications for the clinical utility of kisspeptin‐based therapeutics.

## 4 PATTERNS OF EXPRESSION

The distribution of peripheral and central kisspeptin and its receptor provides important evidence for the pleiotropic functions of kisspeptin beyond the classical reproductive axis.

### 4.1 Peripheral kisspeptin expression

In humans, abundant *KISS1R* mRNA expression has been detected in the placenta and pancreas, as well as lower levels in various other organs, including adipose tissue, heart, kidney, liver, lung, small intestine, spleen, stomach, testis, thymus and fetal lung, as determined by reverse transcription polymerase chain reaction (RT‐PCR).^5^, ^6^, ^7^ In contrast, *KISS1* mRNA has been detected predominantly in the placenta, with the next highest level in the testis, and moderate levels in the liver, pancreas and small intestine.^5^, ^7^

### 4.2 Central kisspeptin expression

#### 4.2.1 Hypothalamic expression

The distribution of kisspeptin‐expressing neurons in the hypothalamus varies in a species‐dependent manner. In rodents, kisspeptin is expressed in two distinct hypothalamic nuclei: the arcuate nucleus (ARC) and the preoptic area (POA) which includes the anteroventral periventricular nucleus (AVPV). Both populations reside near GnRH dendrites and/or cell bodies.^22^, ^23^, ^24^ The ARC kisspeptin neurons coexpress NKB and Dyn to form the so‐called kisspeptin/neurokinin B/dynorphin (KNDy) neurons.^13^ Furthermore, KNDy neurons form abundant close appositions with GnRH neuron distal dendrons with only kisspeptin sufficient to activate the GnRH neuron dendron (despite all three neuropeptides being released from KNDy terminals).^25^ In the human hypothalamus, the two corresponding regions of kisspeptin expression are the infundibular nucleus (equivalent to the rodent ARC) and the rostral POA, with both neuronal populations projecting to GnRH neurons in the mediobasal hypothalamus.^26^, ^27^ The evidence for KNDy neurons in humans is currently less robust than in other species. Whereas putative KNDy neurons have been identified in the infundibular nucleus of women,^28^ ARC kisspeptin neurons in men may not coexpress dynorphin.^29^

#### 4.2.2 Extra‐hypothalamic expression

In rodents, kisspeptin‐immunoreactive fibres have been detected in the amygdala, bed nucleus of the stria terminalis and thalamus,^24^ as well as *Kiss1r* mRNA in the frontal cortex, hippocampus, midbrain, striatum and thalamus.^4^, ^30^ In humans, *KISS1* and/or *KISS1R* mRNA have been identified in the amygdala, caudate nucleus, cerebellum, cingulate gyrus, globus pallidus, hippocampus, medial frontal gyrus, nucleus accumbens, parahippocampal gyrus, putamen, spinal cord, striatum, substantia nigra, superior frontal gyrus and thalamus, as localised by RT‐PCR.^6^, ^7^ Crucially, many of these brain regions form the limbic system, which has important roles in regulating the expression of sexual and emotional behaviours^31^ and provides an anatomical basis that kisspeptin could be involved in these functions.

## 5 THE ROLE OF KNDy NEURONS IN REPRODUCTION

### 5.1 Kisspeptin as the master regulator of the reproductive axis

Preclinical animal models establish that kisspeptin is a potent stimulator of GnRH and gonadotropin secretion in mammals. In the hypothalamic rostral POA of the adult female mouse, dual immunofluorescence experiments reveal that kisspeptin fibres form close appositions with >40% of GnRH cell bodies.^32^ Furthermore, in juvenile male mice, approximately 90% of GnRH neurons express *Kiss1r* mRNA with similar relative expression of *Kiss1r* mRNA observed in GnRH neurons from adult male mice.^33^ Kisspeptin administration stimulates GnRH secretion from in vitro rat hypothalamic explants (but not pituitary fragments),^34^ confirming that kisspeptin stimulates the HPG axis predominantly via the hypothalamus. Central administration of kisspeptin to rats induces expression of *c‐Fos* (a marker of neuronal activation) in GnRH neurons,^35^ and evokes a potent, long‐lasting depolarisation of >90% of GnRH neurons *in situ* in adult mice.^33^ Furthermore, kisspeptin's stimulatory effect has been shown to be abolished by pretreatment with a GnRH antagonist.^22^ Collectively, these observations reveal that kisspeptin sits above GnRH and that GnRH neurons are direct targets for regulation by kisspeptin.

In keeping with in vitro and *in situ* experimental models, central kisspeptin administration can elicit maximal LH secretion across a broad range of species, including rats,^34^ sheep^36^ and monkeys.^37^ Regardless of the administration route, kisspeptin stimulates LH secretion (and to a lesser extent FSH), including following peripheral administration in rodents^34^ and monkeys.^37^ Indeed, these findings are remarkably congruent with *Kiss1* ^*−/−*^ and *Kiss1r* ^*−/−*^ knockout mice, whereby *Kiss1* ^*−/−*^ mutants respond to kisspeptin administration with increased gonadotropin levels, but not *Kiss1r* ^*−/−*^ mutants.^38^ This reveals that kisspeptin acts as the principle endogenous ligand for the kisspeptin receptor. Taken together, these key experimental data underpin the now well‐established control of the HPG axis: kisspeptin stimulates GnRH neurons (residing largely in the POA) in the hypothalamus by activating kisspeptin receptors, which controls the pulsatile and surge release of GnRH into the local hypophyseal‐portal circulation, thereby stimulating the release of downstream pituitary‐gonadal reproductive hormones.^39^ It is interesting to note that species differences in the distribution of GnRH neurons in mammals exists with a significant proportion of GnRH neurons in the lateral anterior tuberal hypothalamus in primates, sheep, ferrets and guinea pigs, compared with a dominance of GnRH neurons in the medial POA and septum in rodents, cats, hamsters, pigs and voles.^40^

### 5.2 Positive and negative sex‐steroid feedback

Ovarian sex‐steroids induce feedback effects on the hypothalamus, in order to modulate the cyclical release of GnRH and gonadotropins.^41^ The feedback actions of oestrogen are mediated through hypothalamic kisspeptin neurons, via oestrogen receptor alpha (ERα),^41^ with a marked proportion of kisspeptin AVPV and ARC neurons coexpressing ERα.^42^, ^43^ Of note, AVPV and ARC kisspeptin neurons display opposing roles in positive and negative sex‐steroid feedback as summarised below:

#### 5.2.1 Positive feedback

Kisspeptin neurons in the rodent AVPV are more abundant in females in whom positive feedback is more significant.^44^ Whereas kisspeptin expression in the mouse AVPV is reduced following ovariectomy, expression increases in response to oestrogen treatment.^42^ In ovariectomised female mice lacking ERα, AVPV kisspeptin expression is unaffected by oestrogen replacement, whereas mice lacking ERβ respond to oestrogen in the same manner as wild‐types,^42^ indicating that ERα (not ERβ) is important for mediating the stimulatory effect of oestrogen on AVPV kisspeptin expression.

#### 5.2.2 Negative feedback

ARC kisspeptin colocalises with NKB (acting at the neurokinin 3 receptor [NK3R]) and Dyn (acting at the kappa‐opioid receptor [KOR]). In this region, kisspeptin neurons of female mice express the *NKB* and *Dyn* genes, as well as the *NK3* and *Dyn* receptor genes.^14^ From a functional perspective, NKB and Dyn demonstrate autoregulation of kisspeptin release, with alternate stimulation of NKB and inhibition by Dyn leading to pulsatile kisspeptin secretion.^45^ Moreover, in addition to negative feedback actions from oestrogen, data in ewes suggests that ARC dynorphin neurons also mediate the negative feedback actions of progesterone by increasing dynorphin release from ARC KNDy neurons to inhibit GnRH and LH pulse frequency.^46^

Together, data from rodent models reveals that oestrogen has positive feedback effects by stimulating kisspeptin expression in the AVPV to induce the GnRH surge, whereas oestrogen has negative feedback effects on the ARC by inhibiting kisspeptin release to suppress GnRH secretion.^47^ In humans and nonhuman primates, the infundibular neurons relays both positive and negative feedback effects with the role of the POA kisspeptin neurons in sex‐steroid feedback undetermined.^26^, ^48^

## 6 THERAPEUTIC APPLICATION IN HUMANS

In view of the data from animal studies revealing kisspeptin's role as a potent inducer of GnRH and gonadotropin secretion, further research attention has been directed towards whether it can stimulate reproductive hormone release in healthy men and women, as well as patients with reproductive disorders due to impaired gonadotropin secretion as detailed below. Furthermore, whether NKB‐signalling  can be manipulated to treat menopausal flushing, endometriosis and uterine fibroids has also been investigated.

### 6.1 Kisspeptin administration stimulates reproductive hormone secretion in healthy men and women

#### 6.1.1 Healthy men

The first study examining kisspeptin administration in humans was undertaken in 2005 using kisspeptin‐54.^20^ In healthy male volunteers, dose‐dependent increases in circulating LH were observed with a 90‐min infusion (0.25–12 pmol/kg/min).^20^ Although FSH and testosterone levels were also increased, the effects were not dose‐dependent.^20^ This is consistent with preclinical animal evidence revealing that kisspeptin's stimulatory effect on LH release is more marked than on FSH in terms of the increase over basal levels.^34^ Additional studies have been conducted to delineate whether shorter kisspeptin isoforms can induce a similar degree of gonadotropin secretion. Administration of kisspeptin‐10 as an intravenous bolus (0.01–3.0 mcg/kg) has been shown to increase circulating LH rapidly and dose‐dependently with peak stimulation at 30 min following 1 mcg/kg.^49^ Moreover, as an infusion, kisspeptin‐10 (4 mcg/kg/h) increased LH secretion four‐fold.^49^ Of note, whereas LH pulses were obscured by this high infusion rate, a lower rate of 1.5 mcg/kg/h not only stimulated LH secretion, but also increased the LH pulse frequency and pulse size.^49^

Given these findings, it is interesting to consider whether administration of different kisspeptin isoforms have equipotent stimulatory responses. Indeed, kisspeptin‐10 and kisspeptin‐54 have been shown in healthy men to have similar effects on gonadotropin secretion when administered intravenously.^50^ In this study, both isoforms were also compared with direct pituitary stimulation using a GnRH infusion. Specifically, GnRH had more potent effects on LH and FSH release: three‐ and two‐fold greater than compared with kisspeptin‐10 and kisspeptin‐54, respectively.^50^ From a therapeutic perspective, although GnRH is more potent, kisspeptin induces a more physiological release of GnRH from the endogenous pool which may be preferable clinically in restoring physiology.^51^

#### 6.1.2 Healthy women

Administration of subcutaneous kisspeptin‐54 (0.2–6.4 nmol/kg) to women in the follicular phase dose‐dependently stimulates LH release, with a response which is seven‐fold greater than that observed on FSH.^52^ Furthermore, a single subcutaneous dose of kisspeptin‐54 (0.15–0.60 nmol/kg) has been shown to temporarily increase LH pulsatility in the follicular phase.^53^ However, it is interesting to consider whether these pulses represent endogenous LH pulses or a pharmacological burst of LH pulses (similar in appearance to endogenous pulses).

Circulating LH increases in all cycle phases following subcutaneous kisspeptin‐54 (0.4 nmol/kg) but the greatest effect occurs in the preovulatory phase (5‐fold greater than in the follicular and luteal phase) and least in the follicular phase.^52^ Similarly, only half of the women in the early follicular phase show clear LH responses following an intravenous bolus of kisspeptin‐10 (0.24 nmol/kg), whereas robust LH responses occur in all women in the luteal and preovulatory phases.^54^ Taken together, these data demonstrate that the response to exogenous kisspeptin depends on the menstrual cycle phase and therefore the prevailing sex‐steroid milieu.

Regarding safety, studies in healthy men and women indicate that acute administration of kisspeptin is well tolerated with no safety concerns.^20^, ^55^ In addition, administration of kisspeptin is not associated with significant changes in heart rate or blood pressure in healthy volunteers, which has important safety implications for the escalating development of kisspeptin‐based therapies.^56^ Moreover, at doses of kisspeptin‐54 known to stimulate gonadotropin secretion, administration has no significant acute or chronic effects on circulating growth hormone, prolactin or thyroid stimulating hormone levels in healthy women.^57^

### 6.2 Kisspeptin administration restores reproductive hormone secretion in hypothalamic amenorrhoea

HA is associated with reduced GnRH pulsatility causing HPG axis suppression and chronic anovulation,^58^ because of excessive exercise, reduced energy intake with weight loss or psychological stress.^59^ Data from rodent models provided the initial evidence that kisspeptin could restore reproductive hormone secretion in women with HA. In prepubertal rats, 72 h of food deprivation was observed to decrease hypothalamic kisspeptin and kisspeptin receptor expression.^60^ Whereas strikingly daily kisspeptin administration from postnatal day 30 to 37 increased the previously suppressed gonadotropin levels.^60^

Turning to women with HA, acute administration of subcutaneous kisspeptin‐54 (6.4 nmol/kg) has been shown to induce an LH response within 4‐h, an effect which is four‐fold more potent than that seen in healthy women in the follicular phase.^61^ However, chronic administration risks tachyphylaxis. In a study examining different administration protocols, twice‐daily subcutaneous kisspeptin‐54 (6.4 nmol/kg) resulted in a progressive diminishment of the acute LH response to the extent that the mean LH fell from 23.3 (±12.1) IU/L (day one) to 1.1 (±0.5) IU/L (day 14).^62^ In contrast, twice‐weekly administration for eight‐weeks produced only partial desensitisation with gonadotropins remaining raised at eight‐weeks,^62^ revealing that tachyphylaxis can be overcome by reducing the administration frequency.

A defining feature of HA is reduced LH pulsatility. It is therefore pertinent that kisspeptin administration not only increases LH secretion, but also restores LH pulsatility in women with HA. During an 8‐h infusion of kisspeptin‐54 (0.01–1.00 nmol/kg/h), both basal and pulsatile LH secretion were temporarily increased.^63^ In fact, in this study the peak number of LH pulses increased three‐fold and the peak LH pulse secretory mass six‐fold compared to placebo.^63^ The observation that continuous infusion of kisspeptin‐54 can restore LH pulsatility may be attributed to several putative mechanisms. Peripheral kisspeptin‐54 may act at GnRH nerve terminals at the median eminence to augment pre‐existing and undetectable GnRH/LH pulses into detectable GnRH/LH pulses. Alternatively, direct or indirect mechanisms may be at play allowing peripheral kisspeptin‐54 to stimulate *de novo* GnRH/LH pulses. Collectively, these studies reveal that kisspeptin administration in women with HA can be used to potently stimulate reproductive hormone secretion and increase LH pulsatility, suggesting similar beneficial effects in other common reproductive disorders.

### 6.3 Kisspeptin administration restores reproductive hormone secretion in hyperprolactinaemia

Hyperprolactinaemia causes HH and infertility due to suppressed LH pulsatility.^64^ Dopamine agonists are highly effective in most cases by normalising prolactin levels and restoring eugonadism.^65^ However, resistance occurs with bromocriptine and cabergoline in approximately 20%–30% and 10% of patients, respectively,^66^ underscoring the need for alternative therapies for resistant and intolerant patients.

Most GnRH neurons do not express prolactin‐receptors,^67^ whereas kisspeptin neurons unequivocally do with hyperprolactinaemia reducing hypothalamic kisspeptin expression.^68^ This reveals that prolactin exerts its suppressive effects on the HPG axis by supressing kisspeptin inputs to GnRH neurons, implying that kisspeptin administration could be used to restore the downstream axis. In a preclinical mouse model of hyperprolactinaemia‐induced anovulation, female mice were observed to have reduced gonadotropin secretion and diminished kisspeptin expression as expected.^69^ Daily administration of kisspeptin‐10 not only restored the biochemical effects of prolactin excess on gonadotropin secretion, but also restored ovarian cyclicity.^69^ Translating these findings into humans, in two women with cabergoline resistant hyperprolactinaemia and chronic amenorrhoea, a 12‐h infusion of kisspeptin‐10 (1.5 mcg/kg/h) induced robust increases in circulating LH and FSH levels, as well as LH pulsatility.^70^ These data highlight kisspeptin as a putative therapeutic target for the management of hyperprolactinaemia, with further clinical studies needed to expand on these exciting preliminary findings.

### 6.4 Kisspeptin administration stimulates reproductive hormone secretion in diabetes‐related hypogonadism

HH occurs in 25%–40% of men with type 2 diabetes mellites.^71^ Although the underlying causative mechanism is almost certainly multifactorial,^72^ it has been posited that suppressed endogenous kisspeptin secretion may link the metabolic and reproductive abnormalities.^73^ In fact, factors decreasing GnRH secretion (such as hyperglycaemia and inflammation) have also been observed to suppress kisspeptin expression in animal models.^73^

In a study investigating acute gonadotropin responses in men with type 2 diabetes (BMI >40 kg/m^2^) and mild biochemical hypogonadism, both an intravenous bolus (0.3 mcg/kg) and continuous 11‐h infusion of kisspeptin‐10 (4 mcg/kg/h) induced two‐ and five‐fold increases in LH secretion, respectively.^74^ In response to intravenous infusion, kisspeptin also increased the LH pulse frequency by 50% and circulating testosterone levels by 35%,^74^ suggesting that kisspeptin administration may serve to enhance endogenous testosterone secretion in men with diabetes. This is relevant given that kisspeptin administration has been observed to enhance glucose‐stimulated insulin secretion in healthy men,^75^ indicating that kisspeptin may modulate both reproductive hormone secretion and insulin release in patients with diabetes with further clinical studies warranted.

### 6.5 Kisspeptin triggers egg maturation in women undergoing in vitro fertilisation

During in vitro fertilisation (IVF), human chorionic gonadotropin (hCG) is used as a surrogate for the mid‐cycle LH surge owing to its structural and biological resemblance to LH.^76^ However, hCG's longer half‐life and more potent effect at the LH receptor^77^, ^78^ can result in prolonged ovarian LH receptor stimulation, causing the potentially life‐threatening complication of Ovarian Hyperstimulation Syndrome (OHSS).^79^ This underscores the need for more physiological stimuli to trigger oocyte maturation.

Following standard superovulation with recombinant FSH and a GnRH antagonist, a single subcutaneous bolus of kisspeptin‐54 (1.6–12.8 nmol/kg) induces an LH surge and dose‐dependently increases egg maturation.^80^ As a result, subsequent studies have investigated whether kisspeptin might provide an alternative trigger for egg maturation in women at high‐risk of developing OHSS. In a randomised trial of high‐risk women, following standard superovulation, women were randomly assigned to receive a single injection of kisspeptin‐54.^81^ At all doses tested (3.2–12.8 nmol/kg), the biochemical pregnancy rate was 63%, clinical pregnancy rate 53% and live birth rate 45%, with no women developing moderate to critical OHSS.^81^

From a therapeutic perspective, the endogenous LH surge lasts 48 h,^82^ compared with 10–12 hours with a single dose of kisspeptin‐54. Given this, the addition of a second dose of kisspeptin‐54 to improve oocyte maturation has been studied. In a randomised, placebo‐controlled trial in women at high risk of OHSS, a second dose of kisspeptin‐54 (9.6 nmol/kg) 10 h following the first dose induced further LH secretion.^83^ This resulted in 71% of women achieving an oocyte yield of ≥60% compared with 45% of women receiving a single dose of kisspeptin‐54.^83^ Together, these studies support the use of kisspeptin administration for oocyte maturation in an IVF setting, including in women at high risk of OHSS. Further clinical studies directly comparing kisspeptin to standard triggers of egg maturation are required.

### 6.6 Generation of kisspeptin analogues

Native kisspeptin has significant potential to treat common reproductive disorders. However, its susceptibility to rapid enzymatic cleavage^84^ and short half‐lives^20^, ^21^ poses specific therapeutic challenges. Moreover, repeated high‐dose administration risks tachyphylaxis,^61^, ^62^ causing diminished stimulation of the HPG axis. To overcome these challenges, synthetic analogues of kisspeptin have been generated, which aims to reduce proteolytic degradation, slow down renal clearance and maintain agonistic activity at the kisspeptin receptor.

Since kisspeptin‐10 is the shortest endogenous isoform capable of activating the kisspeptin receptor, chemical modifications of the kisspeptin‐10 template have produced novel compounds to have increased potency and stability.^85^, ^86^ Once such example is MVT‐602 (previously called TAK‐448). MVT‐602 possesses four replacements [N‐terminal D‐Tyr, Hyp,^47^ azaGly^51^ and Arg(Me)^53^] and one deletion [Asn^46^] at the key protease cleavage sites of kisspeptin‐10,^87^ producing enhanced metabolic stability, potency and water solubility.^85^ Testing of MVT‐602 in healthy men has revealed that when administered as a single subcutaneous bolus (0.001–6 mg) sustained LH stimulation occurs with maximal responses between 6 and 12 h and LH levels remaining raised for 48–72 h.^88^ In contrast, as an initial 0.1 mg subcutaneous bolus followed by a 13‐day infusion (0.01–1 mg per day), MVT‐602 caused an initial surge in circulating LH and testosterone, followed by abrupt reductions by day two. Although these suppressive effects persisted throughout the infusion period, they were reversible after treatment cessation with testosterone levels returning to normal by 14‐days.^88^ Moreover, in a recent study comparing healthy women in the follicular phase with polycystic ovarian syndrome and HA women, subcutaneous administration of MVT‐602 (doses 0.01 and 0.03 nmol/kg) resulted in similar LH increases in the PCOS and healthy groups.^89^ In contrast, MVT‐602 induced an exaggerated and early rise in circulating LH in HA women, with an initial peak at 6.2 h compared with 15.1 h in healthy women.^89^ This can putatively be explained by a greater abundance of kisspeptin receptors in HA leading to an exaggerated gonadotropin rise.

Taken together, generation of kisspeptin analogues offer pharmacological advantages over native kisspeptin with potentially higher and longer‐lasting biological activity, which represents a key advance in the treatment of reproductive disorders. From a clinical perspective, additional studies are needed to optimise the dosing regimens and frequency of administration to stimulate reproductive hormone secretion while preventing tachyphylaxis.

### 6.7 Manipulating neurokinin B‐signalling to treat menopausal flushing

Menopause is the permanent cessation of menstrual cycles following the loss of ovarian follicular activity.^90^ The clinical manifestations related to this decline in ovarian oestrogen production can be distressing and debilitating, including vasomotor symptoms with hot flushes. Given their marked effects on quality of life, more efficacious and safe therapies are warranted, particularly for women with contraindications to standard hormonal pharmacotherapy.^90^

Experimental data implicates the KNDy neurons in the generation of menopausal hot flushes. Menopause results in oestrogen deficiency with loss of negative feedback on KNDy neurons in the ARC/infundibular nucleus. Consistent with this, the infundibular nucleus of ovariectomised monkeys and postmenopausal women are markedly hypertrophied with increased kisspeptin and NKB gene expression.^26^, ^91^ In addition to GnRH neurons, KNDy neurons also project to the medial preoptic area, which is recognised as the central control hub for thermoregulation in mammals, highlighting their role as a key integrative link between the endocrine changes of menopause and vasomotor symptoms. Indeed, ablating ARC KNDy neurons in rats reduces tail‐skin temperature,^92^ whereas subcutaneous injections of an NK3R agonist (senktide) acutely increases tail‐skin temperature in ovariectomised mice.^93^ Furthermore, selective activation of ARC kisspeptin neurons has been observed to induce a hot‐flush‐like vasodilation response in both female and male mice.^94^ Additionally, in this study, brief activation (for 2‐min) of ARC kisspeptin axon terminals in the preoptic area recapitulated this heat‐dissipation response in female mice.^94^ Conversely, pre‐treatment with a cocktail of neurokinin receptor antagonists (given that NKB has a degree of affinity for all three neurokinin receptors) delivered to the preoptic area prevented the increase in tail‐skin temperature following activation of ARC kisspeptin neurons.^94^ Moreover, a very recent study examined the effects of administration of a peripherally restricted kappa receptor agonist (which can access brain areas including the ARC via the fenestrated capillaries of the median eminence) on vasomotor symptoms.^95^ In this study, inhibition of kisspeptin neuronal activity by chronic administration of kappa opioid receptor agonists to bilaterally ovariectomised mice with experimentally induced hyperactivity of KNDy neurons significantly reduced the elevated body temperature.^95^ Taken together, this series of preclinical experiments emphasises the importance of KNDy neurons in the generation of hot flushes, as well as the promise of targeting these neurons to treat vasomotor symptoms.

Translating these rodent models into clinical studies, an intravenous infusion of NKB to healthy premenopausal women has been observed to induce hot flushes that are typical in location and duration to those experienced by postmenopausal women, as well as cause significant elevations in skin temperature and heart rate.^96^ This supports the rationale for pharmacological blockade of NKB as a novel therapeutic target in menopausal hot flushes, which has triggered several studies. In a phase 2, randomised, double‐blind, placebo‐controlled study of 28 healthy women aged 40–62 years with severe hot flushes, oral administration of an NK3R antagonist (MLE4901, 40 mg twice daily for 4‐weeks) reduced the number of hot flushes by 45% compared to placebo.^97^ In this study, no serious adverse events occurred.^97^ Three participants developed a mild and transient rise in liver transaminases following MLE4901 which returned to baseline.^97^ Additional analysis reveals that the symptomatic improvement following MLE4901 occurred within 3 days of treatment and persisted throughout the duration of the study.^98^ In keeping with these findings, administration of a further NK3R antagonist (fezolinetant) to 352 women with moderate/severe vasomotor symptoms has been observed to produce a rapid reduction in symptom frequency.^99^ The most common treatment emergent adverse events in this study were nausea, diarrhoea, fatigue, urinary tract infections, sinusitis, headache and cough.^99^ Nine participants developed a mild and transient rise in liver transaminases following fezolinetant treatment typically between 4 and 8 weeks of treatment.^99^ In a further study, administration of a dual NK1 and NK3 receptor antagonist (elinzanetant: NT‐814), blocking the endogenous effects of Substance P and NKB, respectively, has been observed in postmenopausal women with moderate/severe hot flushes to result in a rapid and marked improvement in hot flushes and waking due to night sweats.^100^ The most frequent treatment‐related adverse events were mild somnolence and headache, which occurred most regularly in the highest treatment group (300 mg daily).^100^ Taken together, these promising results indicate the benefit of novel agents targeting the KNDy system, with a range of compounds now in late‐phase development.

### 6.8 Manipulating neurokinin B‐signalling to treat uterine fibroids and endometriosis?

Uterine fibroids and endometriosis are highly prevalent conditions which affect up to 25% and 10% of women, respectively.^101^, ^102^ Physiological oestrogen levels provide a hormonal drive to the endometrium and myometrium; therefore, standard medical management relies on downregulation of the HPG axis using GnRH agonists and antagonists.^103^, ^104^ However, they are associated with undesirable short‐term hypoestrogenic and vasomotor symptoms, as well as long‐term detrimental effects on bone health and cardiovascular risk.^105^, ^106^ It is accepted that maintaining oestradiol levels within a therapeutic target of 30–50 pg/ml (i.e., 110–184 pmol/l) reduces the hormonal drive to the endometrium and myometrium while avoiding the adverse effects associated with current therapies.^107^

A recent study investigated the effects of NK1 and NK3 receptor antagonism (elinzanetant: NT‐814) on reproductive hormone secretion across two consecutive menstrual cycles in 33‐healthy premenopausal women.^108^ Following a baseline assessment cycle, women in the second cycle were randomised to once daily oral elinzanetant (40–120 mg) or placebo. This revealed that elinzanetant reduced oestradiol and progesterone levels in a dose‐dependent manner without causing vasomotor symptoms. From a therapeutic perspective, the highest dosing arm was observed to lower circulating oestradiol to potentially ideal levels for uterine fibroids and endometriosis.^108^ Regarding safety, elinzanetant was well tolerated with no safety concerns identified during the study, nor any clinically relevant changes in blood pressure, heart rate or electrocardiogram parameters.^108^ Therefore, further studies are now required in women with hormone driven disorders to examine the effects of NK1 and NK3 receptor antagonism.

## 7 CONCLUSION

The neuropeptide kisspeptin has fundamental importance in mammalian reproduction, with a plethora of experimental animal models demonstrating its significance as the master regulator of GnRH and gonadotropin release. Moreover, outside of the classical reproductive axis, kisspeptin has increasingly established roles, including in reproductive behaviour,^109^ metabolism,^110^ bone biology^111^, ^112^ and cognition,^113^ with imminent studies undoubtedly pending to dissect these functions in more detail (particularly in humans). The exciting reproductive observations in animal models has triggered significant interest in targeting the kisspeptin‐pathway to treat human reproductive disorders. Across an increasing number of studies, kisspeptin has been shown to restore reproductive hormone secretion in men and women with hypogonadism (such as HA, hyperprolactinaemia and diabetes‐induced hypogonadism), as well as safely induce egg maturation in women undergoing IVF. Clinical studies have shown that acute administration of kisspeptin is safe and well tolerated in both healthy volunteers and patient groups, with further evidence from studies employing chronic protocols needed to confirm safety over protracted periods of time. Through modification of native kisspeptin templates, the generation of kisspeptin analogues with enhanced potency and stability has prospect of further exploiting the clinical utility of kisspeptin‐based therapeutics. In addition, emerging therapeutic roles based on neurokinin B‐signalling for the management of menopausal flushing, endometriosis and uterine fibroids are increasingly recognised. To this end, kisspeptin and neurokinin B offer exciting translational potential for novel therapies for reproductive health.

## AUTHOR CONTRIBUTIONS

**Edouard G Mills:** Conceptualization; investigation; writing – original draft; writing – review and editing. **Waljit S Dhillo:** Conceptualization; investigation; writing – original draft; writing – review and editing.

## CONFLICT OF INTEREST

EGM has nothing to declare. WSD has undertaken consultancy for Myovant Sciences Ltd and KaNDy Therapeutics.

## Data Availability

N/A
